# Divergent Solutions to Visual Problem Solving across Mammalian Species

**DOI:** 10.1523/ENEURO.0167-18.2018

**Published:** 2018-07-11

**Authors:** Faiz Mustafar, Michael A. Harvey, Abbas Khani, József Arató, Gregor Rainer

**Affiliations:** 1 Visual Cognition Laboratory, Department of Medicine, University of Fribourg, Fribourg 1700, Switzerland; 2 Department of Neuroscience, School of Medical Sciences, Universiti Sains Malaysia, 16150 Kubang Kerian, Kelantan, Malaysia; 3 Department of Fundamental Neuroscience, University of Geneva, Geneva 1205, Switzerland; 4 Department of Cognitive Science, Central European University, Budapest 1051, Hungary

**Keywords:** behavior, comparative learning, monkey, operant conditioning, tree shrew, vision

## Abstract

Our understanding of the neurobiological underpinnings of learning and behavior relies on the use of invasive techniques, which necessitate the use of animal models. However, when different species learn the same task, to what degree are they actually producing the same behavior and engaging homologous neural circuitry? This question has received virtually no recent attention, even as the most powerful new methodologies for measuring and perturbing the nervous system have become increasingly dependent on the use of murine species. Here, we test humans, rats, monkeys, and an evolutionarily intermediate species, tree shrews, on a three alternative, forced choice, visual contrast discrimination task. As anticipated, learning rate, peak performance, and transfer across contrasts was lower in the rat compared to the other species. More interestingly, rats exhibited two major behavioral peculiarities: while monkeys and tree shrews based their choices largely on visual information, rats tended to base their choices on past reward history. Furthermore, as the task became more difficult, rats largely disengaged from the visual stimulus, reverting to innate spatial predispositions in order to collect rewards near chance probability. Our findings highlight the limitation of muridae as models for translational research, at least in the area of visually based decision making.

## Significance Statement

Research into the biology of cognition and behavior increasingly relies on studies performed in murine species (mice and rats). This is due to the explosion of powerful new, murine specific, genetic tools allowing for unprecedented control in imaging and manipulation of neural circuit elements. Lacking, are concomitant comparative behavioral studies necessary toward translating these exciting results to other species, particularly humans. We show here that, unlike primates, muridae incorporate species-specific predispositions and bias into their behavioral strategy toward the solution of a simple visual task and are therefore engaging specific circuits not used by primates during task performance. This contradicts the long-held idea that learning abolishes species-specific predispositions and highlights the importance of comparative behavioral studies in translational neuroscience.

## Introduction

**Figure 1.**
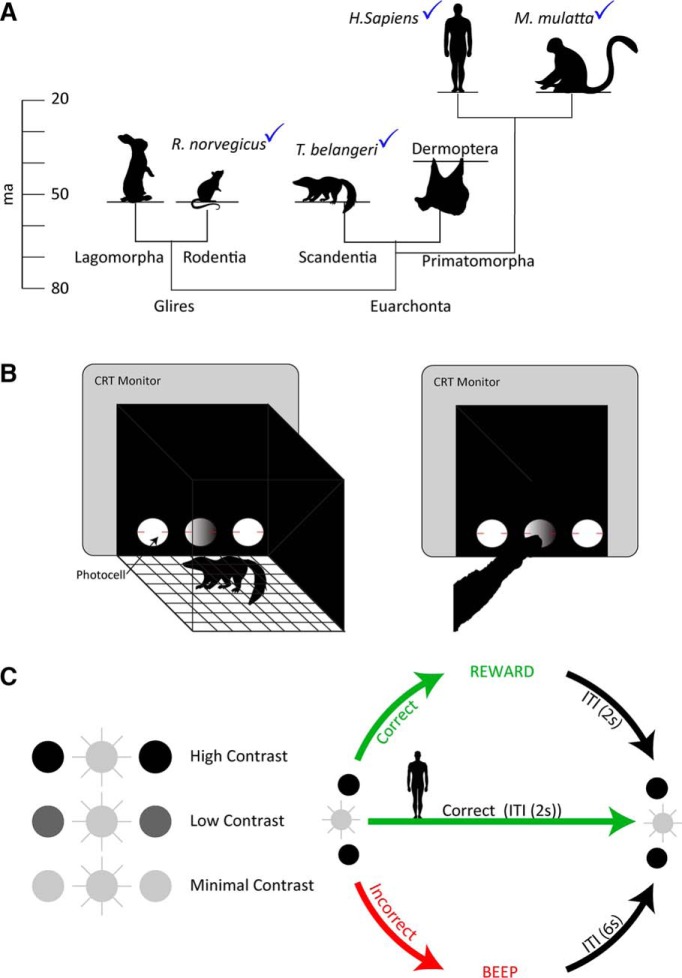
Experimental setup. ***A***, The approximate times of divergence for the three taxonomic orders studied (Springer et al., 2003). ***B***, Rats and tree shrew training took place in an identical test chamber, where a nose poke interrupted a photocell indicating a response. The same stimulus/response panel was used for the monkeys, except they were seated in a primate chair and used their hand to make a response. ***C***, The stimulus configuration consisted of a flickering (15 Hz) target and two constantly illuminated distractors. Initially, in the high-contrast condition, the luminance of the distractors was much lower than that of the target. Following acquisition, animals were transferred to progressively more difficult contrast conditions until they received final training in the minimum-contrast condition. Note that the luminance of the target never changed.

Whether or not different animals use their brains in the same way when learning a particular task is an important question relating to translational research (Benatar, 2007; Shanks et al., 2009; Nestler and Hyman, 2010; Huberman and Niell, 2011; Baker, 2013; Hale, 2014), because the degree to which it applies limits the applicability of results obtained in one mammalian species to others and particularly to humans. Addressing this issue requires the careful analysis of behavioral performance and strategy across different species performing an identical behavioral task, an effort that has received virtually no attention particularly in the last few decades (Thorndike, 1911; Spence, 1936; Seligman, 1970; Masterton and Skeen, 1972; Bitterman, 1975; Markowitsch and Pritzel, 1977; Macphail, 1987). Here, we test two diurnal mammalian species, monkeys (*Macaca fascicularis*) and tree shrews (*Tupaia belangeri*), and one nocturnal mammalian species, rats (*Rattus norvegicus*), on an identical visual discrimination task (Fig. 1). *M. fascicularis*, or crab eating macaques, are social, diurnal old-world monkeys native to Southeast Asia. They represent a popular laboratory model for studies of the visual system as their perceptual abilities closely match those of humans, from which they diverged ∼25 million years ago (Ma; Stewart and Disotell, 1998; Janecka et al., 2007). *T. belangeri*, or northern tree shrews, are also diurnal and native to Southeast Asia but are asocial. Their phylogenetic order has seen several revisions from insectivora, to primata, and finally to Scandentia, which is thought to have diverged from the primate/dermoptera line between 60 and 70 Ma (Roberts et al., 2011). While not as popular as rodents and primates for studies of the visual system, they nevertheless represent an important model due to their relatively close phylogenetic relationship to primates, manifested in part by a similar visual apparatus. Unlike the macaques and tree shrews, *R. norvegicus* or Norway rats are primarily nocturnal. They are social rodents and represent the other side of the Eurochonta/Glires split, which is thought to have occurred some 80 Ma (Roberts et al., 2011). Since detection of rapidly changing environmental features is of importance for all three species, we chose to investigate their ability to discriminate a 15-Hz flickering light stimulus from two continuously illuminated distractors under three contrast conditions.

## Materials and Methods

The local ethical committee on animal experimentation (canton of Fribourg), approved all experimental procedures.

### Subjects

Five humans of either sex (*Homo sapiens*), two male macaque monkeys (*M. fascicularis*), six tree shrews of either sex (*T. belangeri*), and four male Long Evans Hooded rats (*R. norwegicus*) participated in the experiments.

### Apparatus

Tree shrew and rat behavioral training took place in a 38 × 35.5 × 36 cm (width × height × depth) arena. The arena walls were made of matte-black plastic panels. Three equidistant (4 cm) nose pokes (6.5 cm in diameter) equipped with photocells, were located 3.5 cm from the floor at the front of the arena. Nose pokes were open at the back such that visual stimuli could be delivered through the nose poke via a 19-inch CRT monitor with a refresh rate of 120 Hz (Fig. 1*B*). A small rectangular reward container (4.5 × 6 cm) equipped with a pair of LEDs was placed at the back, opposite to the middle nose poke. A speaker and a camera were attached on top of the chamber for feedback and monitoring purposes. Food rewards were delivered via a pedestal mounted pellet dispenser (Med Associates).

A similar experimental setup was used for the monkeys, except that the monkeys were tested in a wooden monkey box instead of the arena (for illustration, see Mustafar et al., 2015), which was equipped with a camera and a speaker. The monkeys were seated in a custom-made primate chair, with no head fixation. An opening on the front right side of the chair allows the monkey’s right hand to engage with the task by using its hand to interrupt the photocells in the same nose poke holes as used by the rats and tree shrews. This chair was fixed in the monkey box, 40 cm in front of the stimuli panel. The stimulus panel was identical to that used for tree shrew (Fig. 1*B*) and rat testing and was mounted on a custom-made steel stand, backed with a computer monitor. Food rewards were delivered via a plastic tube. All animal subjects were habituated to the experimental setup before the experiment described here and trained to nose/hand poke for food reward in the absence of visual stimulation. Humans were tested using the same visual stimuli with responses registered by a key press.

### Behavioral training

**Figure 2.**
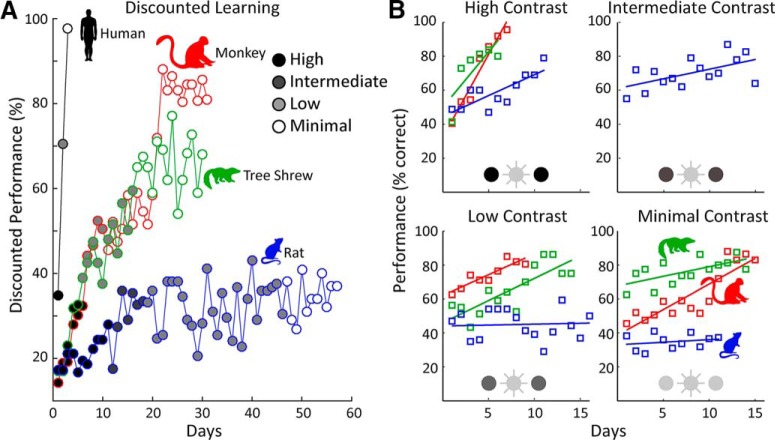
Learning dynamics. ***A***, To facilitate visual comparison between species, we calculated the discounted performance (see text). The results for a representative animal from each species is shown for the four contrast conditions used. The shading of the markers denotes contrast condition. Note, only rats received training in the intermediate-contrast condition. ***B***, The nondiscounted learning curves for the same animals, except the humans, are shown for the four contrast conditions. The slopes of the linear regression lines plotted in ***B*** provide a measure of learning rate.

All nonhuman animals were maintained on a 12/12 h light/dark cycle with water available *ad libitum*, under light food deprivation. Specifically, animals had no access to food during their rest periods, and the task performance represented their first access to food (45-mg flavored pellets) during the day, which was supplemented with additional food pellets following task performance. All experiments were conducted in a dark environment. Subjects were trained on daily sessions to discriminate the flicker stimulus from the stationary stimulus and were allowed to work until satiated (>10 min without a response). Note that learning rate depends not only on the number of training days but also on the number of trials per day. For example, during the initial learning in the high-contrast condition, monkeys performed on average 155 trials per day, ∼60% more than tree shrews (*n* = 96) and rats (*n* = 98). This means that learning rate estimated by day (Fig. 2*B*) is certainly valid for comparing rats and tree shrews, while we may in fact be slightly overestimating learning capacities of the monkey subjects at least during initial learning. However, considering all contrast conditions, rats in fact performed significantly, ANOVA [*F*_(2,9)_ = 251, *p* < 1e-7], more trials (M = 7097, SEM = 178) than either monkeys (M = 6225, SEM = 125, *p* < 0.05) or tree shrews (M = 2755, SEM = 128, *p* < 1e-7), where M is the mean and SEM the standard error of the mean. Indeed, already at the point of maximum performance in the high-contrast condition, rats had performed no fewer trials than monkeys unpaired *t* test (*t*_(4)_ = 2.1, *p* > 0.1). To the extent that the number of completed trials reflects the motivation of the animals, we can thus conclude that tree shrews and rats were equally motivated throughout training. Visual stimuli consisted of one target and two distractors. The target stimuli flickered at a constant rate of 15 Hz and had a luminance intensity of 36 cd/m^2^. The distractors did not flicker and their luminance intensity varied according to the three experimental conditions: high contrast (4.5 cd/m^2^), low contrast (18 cd/m^2^), and minimum contrast (36 cd/m^2^). In each trial, visual stimuli were presented simultaneously with one target and two distractors appearing randomly at the three possible locations. Nonhuman subjects were rewarded with food pellets for each correct response and a beep sound was presented with an extended intertrial interval for each incorrect response (Fig. 1). Visual stimuli were presented using MATLAB (MathWorks) and Psychtoolbox 3 (Brainard, 1997), on a Compaq 19-inch CRT monitor, calibrated for linearity with a Minolta TVCA-II color analyzer. We adjusted the number of pellets delivered for correct trials according to species, such that monkeys received multiple (2–3) 45-mg pellets to account for their greater body weight compared to rats and tree shrews. Two additional stimulus conditions were used only for the rats. First, as rat performance was trailing the other species after the high-contrast condition, an intermediate-contrast (9 cd/m^2^) condition was introduced. Second, as rats failed to learn the low-contrast condition, they received randomly interspersed trials of high, low, and intermediate contrast during the minimum-contrast condition.

## Results

Monkeys, tree shrews, and rats were trained on an identical visual discrimination, to detect a flickering (15 Hz) stimulus in the presence of two distractor stimuli. In an initial condition, high contrast, the luminance of the distractor stimuli was lower than that of the flickering stimuli.

Following acquisition in the high-contrast condition, animals were moved to low-contrast and then to minimum-contrast conditions. Due to their relatively poor performance on the high-contrast condition, rats received training on an additional, intermediate-contrast, condition (see Materials and Methods). Details of the experimental set up and design can be seen in Figure 1*B*,*C*.

To facilitate the comparison of learning dynamics between the different species, we computed the discounted performance, Dp=PcLt/Ld, where *Dp* = discounted performance, Pc = percentage correct, Ld = distractor luminance, and Lt = target luminance. The results for all species are shown in Figure 2*A*. Monkeys exhibited the highest overall performance with a ceiling of ≈85% correct. Tree shrews exhibited a similar learning dynamic but had a lower ceiling, ≈70%. Rats were slower to learn, showed a lower ceiling, and largely failed to learn under the more challenging conditions. Predictably, humans outperformed the other species.

**Figure 3.**
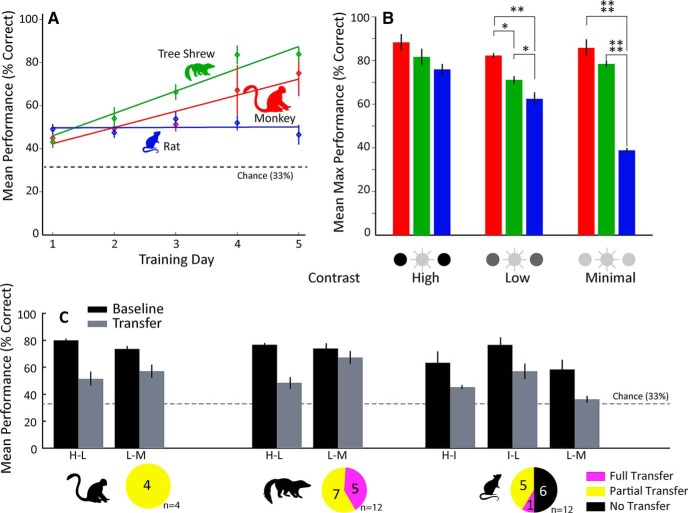
Behavioral performance. ***A***, Mean performance over the first 5 d of training for all species shown. Initially, all species are at a similar level of performance and begin to diverge on day 3. At day 5, the monkeys and tree shrews are still equivalent, while the rats have fallen behind. ***B***, We calculated the maximum performance for each condition by averaging the best 3 d for each animal for that condition. Asterisks denotes significance (*p < 0.05, **p < 0.01, ****p < 0.001). ***C***, To quantify transfer, we compared the mean of the final three sessions of the prior contrast condition to the first three sessions of the subsequent contrast condition. Mean and SEM performance values are shown for a representative animal of each species. The pie charts illustrate the number of cases of transfer observed in that species, i.e., the number of transfer conditions multiplied by the number of subjects for each species. For statistics and description of transfer conditions, see text.

To quantify this, we first calculated the linear regression of performance over the first 5 d of training in the high-contrast condition, which represents the first 5 d of discrimination learning for all species. Note that animal subjects tended to respond above chance (33%) even on the first day of training, likely because of the high salience of the flickering stimulus and their familiarity with the training apparatus (see Materials and Methods). An example from a representative animal of each species is shown in Figure 2*B*. Using this metric a one-way ANOVA showed a main effect of species on learning rate [*F*_(2,9)_ = 23.93, *p* < 0.001], and *post hoc* (Tukey’s HSD) analysis revealed that monkeys (*p* < 0.05) and tree shrews (*p* < 0.01) learned more quickly than did the rats (Fig. 3*A*). There was no significant difference in learning rate between the monkeys and tree shrews during this period. Next, we defined maximum performance as the mean of the three highest performance sessions in each condition (Fig. 3*B*). For the high-contrast condition, a one-way ANOVA showed no effect of species on maximum performance [*F*_(2,9)_ = 1.81, *p* > 0.1]. A main effect of species was found for the low-contrast condition [*F*_(2,9)_ = 12.15, *p* < 0.01], and *post hoc* analysis now showed monkeys doing significantly better than both tree shrews (*p* < 0.05) and rats (*p* < 0.01) and tree shrews performing better than the rats (*p* < 0.05). For the minimal contrast condition, there was also a main effect of species [*F*_(2,9)_ = 173.2, *p* < 1e-7]; monkeys and tree shrews did not differ from each other while both species outperformed rats (*p* < 1e-5).

In short, monkeys and tree shrews learned more quickly than did the rats, and monkeys and tree shrews tended to achieve higher maximum performance than did the rats, with these differences becoming more pronounced as the task became more difficult.

### Transfer

We next wanted to know how capable the different species were at using information about the previous task condition toward performance in the subsequent, more difficult condition. We compared performance, percentage correct, in each individual animal on the last three sessions of the prior condition against the first three sessions of the subsequent condition (transfer) with no difference (*p* > 0.05) indicating full transfer (Fig. 3*C*). If there was a significant decrease in performance using this measure, performance in the transfer condition was tested against chance performance, which was set to 33%. If the animals did significantly better than chance, we considered this as partial transfer. For each species, we thus assessed transfer in a total number of cases, i.e., number of transfer conditions multiplied by number of animals (Table 1; Fig. 3*C*). Note that monkeys and tree shrews always displayed some evidence of transfer, whereas rats failed to show any evidence of transfer on half of the analyzed cases. Examining interindividual differences, we observed that most tree shrews exhibited full transfer in at least one of the transfer conditions, whereas animal TS3 only showed partial transfer in both conditions. Transfer from high to low (H-L) contrast conditions appeared more difficult with only one of the six animals showing full transfer, whereas four of the six animals showed full transfer in the low to minimal (L-M) condition. Interestingly, animal TS1 actually significantly improved in the L-M contrast condition, maybe because TS1 only achieved 46% correct pretransfer performance and quickly achieved higher levels of performance at minimal contrast. Transfer performance of rats was highly dependent on contrast condition. In the first two transfers, high to intermediate (H-I) and intermediate to low (I-L), all rats showed at least some evidence of transfer, with animal R2 even achieving full transfer in the H-I condition. For the low to minimal (L-M) contrast condition, none of the rats exhibited any significant transfer. Note that the rats’ transfer estimate benefits from the inclusion of the intermediate-contrast condition, and we may thus be slightly overestimating their transfer abilities relative to the other two species that transitioned directly from high to low contrast.

**Table 1.** Transfer learning statistics

Case	M*_*1*_*(SEM)	M_2_ (SEM)	T_1_ (df = 2)	T_2_ (df = 2)	p1	p2
M1 H-L	91 (2.9)	68 (3.4)	41.2	21.6	0.0006*	0.002*
M2 H-L	83 (0.8)	54 (5)	14.7	8.7	0.004*	0.01*
M1 L-M	81 (2.5)	48 (1.9)	68.7	16.9	0.0002*	0.003*
M2 L-M	77 (1.8)	59 (4.7)	8	12	0.01*	0.007*
TS1 H-L	82 (7)	50 (4.12)	6.2	8.7	0.02*	0.01*
TS2 H-L	79 (7.3)	71 (2.6)	2.9	31.5	0.1	0.001*
TS3 H-L	82 (8.7)	61 (3.5)	7.6	16.9	0.01*	0.003*
TS4 H-L	81 (4.3)	48 (4.2)	14.8	7.7	0.004*	0.01*
TS5 H-L	85 (4.9)	42 (0.3)	19.3	69.8	0.002*	0.0002*
TS6 H-L	82 (1.1)	52 (4.1)	18.6	9.6	0.003*	0. 01*
TS1 L-M	46 (2.5)	61 (2.1)	-10.4	28.5	0.009*	0.001*
TS2 L-M	76 (3)	56 (4.4)	27.8	11.3	0.001*	0.007*
TS3 L-M	78 (3.8)	66 (0.8)	5.7	82.7	0.03*	0.0001*
TS4 L-M	65 (4.9)	64 (5.6)	0.2	11.9	0.9	0.007*
TS5 L-M	63 (3.6)	64 (1.2)	-0.43)	54.2	0.7	0.0003*
TS6 L-M	79 (3.8)	72 (4.6)	1.8	17.7	0.2	0.003*
R1 H-I	67 (2)	54 (10.7)	3.1	4.2	0.09	0.053
R2 H-I	75 (2.9)	74 (3.2)	0.22	27.6	0.81	0.001*
R3 H-I	74 (3.2)	53 (12.3)	2.9	3.5	0.1	0.07
R4 H-I	63 (8)	43 (1.15)	4.6	22	0.04*	0.002*
R1 I-L	82 (2.6)	44 (4.8)	13.4	5.0	0.005*	0.03*
R2 I-L	74 (5.9)	47 (2.2)	7.2	13.9	0.02*	0.005*
R3 I-L	70 (8)	53 (5.2)	6.1	7.9	0.03*	0.015*
R4 I-L	76 (5.2)	57 (5.4)	5	9.4	0.04*	0.01*
R1 L-M	52 (0.5)	31 (3.4)	11.2	-1	0.008*	0.4
R2 L-M	46 (2.8)	30 (3.5)	7	-1.8	0.02*	0.2
R3 L-M	49 (5.2)	29 (5.13)	4.1	-1.6	0.056	0.2
R4 L-M	58 (6.8)	36 (2.1)	9.3	3.1	0.01*	0.09

The mean behavioral performance pre- and post-transfer (M_1_,M_2_) and related SEM values are shown for all transfer cases for each individual animal of all three species (M, monkey; TS, tree shrew; R, rat) and transfer condition (H, high contrast; I, intermediate contrast; L, low contrast; M, minimal contrast); i.e., row TS4 H-L provides information on tree shrew 4 on the transfer from high- to low-contrast condition. Statistics for paired *t* tests are shown for *t* test T1, testing M_1_ against M_2_, as well as *t* test T2, testing M_2_ against chance performance (33%). Triangles denote transfer type (compare Fig. 3C), with full, partial, and no transfer denoted by purple, yellow, and gray, respectively.

### Spatial bias index

**Figure 4.**
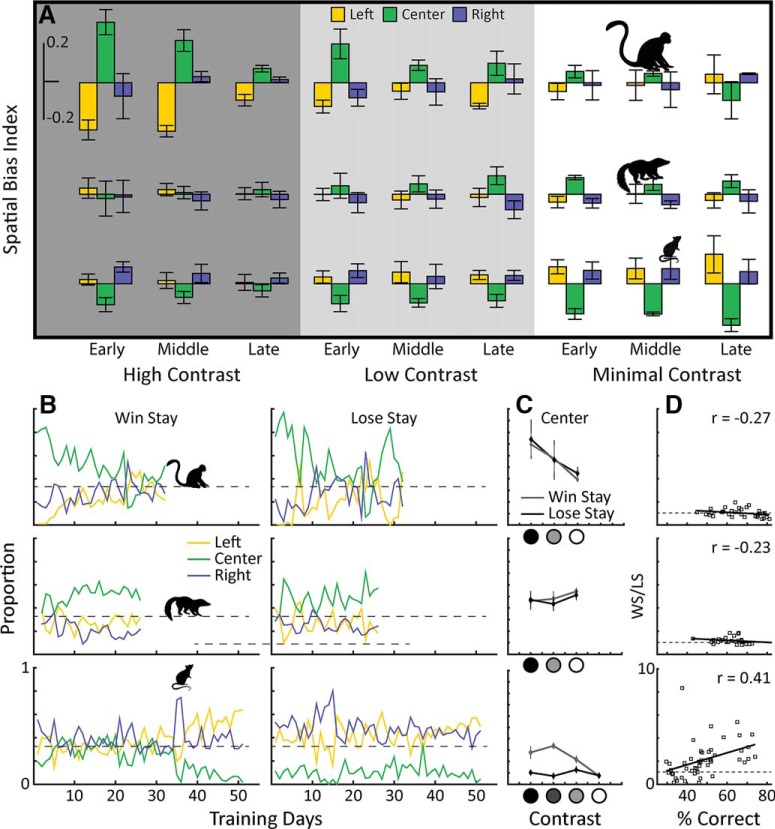
Behavioral strategy. ***A***, The spatial bias index (see text) for the three species is shown for the three contrast conditions. ***B***, The probability of remaining at the three response locations following a reward, or following no reward is shown for each species and training stage. ***C***, The mean values for win stay and lose stay for the center position over the different contrast conditions are shown. ***D***, Ratio of win stay/lose stay (WS/LS) versus behavioral performance at the center position for each of the species. Only for rats is WS/LS ratio correlated with behavioral performance, suggesting that they are able to sustain good behavioral performance using reward to counteract their spatial bias against the center nose poke.

To examine potential differences in learning strategy, we looked at how the different species distributed their responses across the three nose/hand pokes at different stages of the learning process, as well as across the contrast conditions. Since the target was presented pseudo-randomly at three positions, small deviations from an equal distribution may occur. We took this into account using the following equation to calculate a spatial bias index for each location, ${SBI}_{loc}={(Nr}_{loc}-{Nt}_{loc})/N$. Where ${Nr}_{loc}$is the number of times the animal responded at a given location, $Nt_{loc}$is the number of times the target actually appeared at that location, and N is the total number of trials. Thus, positive and negative SBI values indicate preference and avoidance, respectively, for a given location. We then defined training stage as either early, middle, or late by dividing each contrast condition into equal thirds (Fig. 4*A*). Monkeys initially showed a strong center bias, mainly at the expense of the left response location. This bias decreased as performance increased in the high-contrast condition, reappeared on transfer to the low-contrast condition, and finally disappeared entirely. A two-way ANOVA showed that, for the monkeys, both training stage and contrast condition contributed to the center bias [*F*_(2,9)_ = 7.21, *p* < 0.05; *F*_(2,9)_ = 5.07, *p* < 0.05].

Unlike the monkeys, tree shrews distributed their responses equally between the three nose pokes, and there was no significant effect of training stage on response bias [*F*_(2,45)_ = 0.33, *p* > 0.5]. However, the tree shrews did develop a small center bias in the more difficult contrast conditions, and this was reflected by a significant effect of contrast condition [*F*_(2,45)_ = 4.97, *p* < 0.001]. Rats tended to avoid the center nose poke early in training, and while this center avoidance was attenuated during acquisition under the high-contrast condition, the effect of training stage was not significant [*F*_(2,36)_ = 1.66, *p* > 0.1]. Unlike the monkeys, the rats spatial bias, manifested by a center avoidance, became more and more pronounced in successive contrast conditions, and this was highly significant [*F*_(3,36)_ = 21.11, *p* < 1e-3]. In summary, monkeys showed an initial center bias, which was largely reduced during training, tree shrews initially showed no bias, but developed a small center bias toward the end of training, and rats tended to avoid the center, which became especially pronounced in the more difficult conditions.

### Decision making strategy

To better understand what was driving the animals toward or away from the center location, we examined the probability of an animal making a 2nd response at the position where they had just been rewarded, a “win stay” strategy. We compared this to the probability of making a 2nd response at a location where they were not rewarded, a “lose stay” strategy (Fig. 4*B*). Specifically, the number of returns to a given location, either following a reward or following no reward, is divided by the total number of visits to that location to form our win stay and lose stay ratios, respectively. Using this metric, the behavior of all three species at the central position clearly differs from the two flanking positions. Both monkeys and tree shrews were more likely to return to central position than to the flanking positions regardless of reward history, that is, they had the tendency to perseverate at the center position. Rat behavior, however, deviated from that of the monkeys and tree shrews. If a response at the center location went unrewarded, the rats almost never returned on the following trial, a lose-leave strategy that persisted throughout all contrast conditions. Conversely, following a reward at the center position, the rats were equally likely to return there as not. As the task became more difficult, this disparity diminished until the rats became equally unwilling to revisit the center position regardless of reward history. In Figure 4*C*, we compare the proportion of win stay and lose stay responses for the three species over the different contrast conditions. For monkeys and tree shrews, there was never a significant difference in these measures. For the rat during the high-contrast condition win stay was significantly more likely (M = 0.28, SEM = 0.5) than lose stay (M = 0.1, SEM = 0.01), *T*_(6)_ = 3.2, *p* < 0.05. The same was true for the intermediate-contrast condition: win stay (M = 0.34, SEM = 0.03), lose stay (M = 0.07, SEM = 0.03), *t*_(6)_ = 6.9, *p* < 1e-3, but not for low contrast: win stay (M = 0.22, SEM = 0.03), lose stay (M = 0.13, S = 0.03), *t*_(6)_ = 2.0, *p* = 0.09, nor for minimum contrast: win stay (M = 0.07, SEM = 0.01), lose stay (M = 0.08, SEM = 0.01), *t*_(6)_ = -0.2, *p* > 0.8. Here, the rat appears to be overcoming its spatial bias against the center enough to increase its performance. Finally, in Figure 4*D*, we compare the win stay/lose stay ratio with behavioral performance. Somewhat counterintuitively, better performance in the rat is positively correlated with this ratio (*r* = 0.41, *p* < 0.01). This is not the case for the monkeys and tree shrews, (monkey, *r* = -0.27, *p* = 0.15; tree shrew, *r* = -0.23, *p* = 0.25).

## Discussion

We used a visual discrimination task to examine the learning related behavioral characteristics in different mammalian species. Animals were trained to discriminate flickering target stimuli from nonflickering distractors in different contrast conditions.

### Learning rate and performance

Monkeys and tree shrews showed a similar learning dynamic across all contrast conditions, whereas rats initially learned the task at a lower rate and failed to acquire the task in the more challenging conditions. The rats’ failure to learn is perhaps surprising given that all species are capable of detecting flicker stimuli at 15 Hz (Shumake et al., 1968; Schechter and Winter, 1969, 1971; Williams et al., 1985; Callahan and Petry, 2000) consistent with their initial above chance performance in the high-contrast condition. Our data indeed support the idea that all animals were initially reliant to some degree on contrast cues, as all at some point showed a significant drop in performance on transfer from the higher contrast conditions. However, unlike the monkeys and tree shrews, rats benefited less from training under the prior condition when moved to a lower contrast condition, i.e., they exhibited less evidence of transfer. Perhaps this indicates that whereas the monkeys and tree shrews were using both contrast and flicker, the rats were much more reliant on the contrast. Second, we deliberately used a very light food deprivation schedule. This was done as more strict deprivation may fundamentally alter the way in which an animal learns a task, a potential confound for translational studies (Moran, 1975). Perhaps, under mild deprivation, the rats were content to collect rewards on a random basis when the task became more challenging. Notably the animals did not simply stop working, in fact for the rats, the overall number of trials per day were similar between the four contrast conditions.

### Response bias

Response bias impedes performance on sensory decision tasks, because animals are relying on internal predispositions rather than basing their behavior on sensory input. Response bias thus generally decreases during learning, allowing animals to maximize rewards and achieve high performance levels (Krechevsky, 1932; Harlow, 1950; Levine, 1959). This is exactly the behavior shown by the monkeys. During learning in the high-contrast condition, monkey bias gradually decreased as performance improved, a pattern that was repeated in the low-contrast condition. While a similar pattern of behavior was seen in the rat during the high-contrast condition, they showed the opposite trend in the more difficult conditions, with a strong bias emerging and persisting throughout the rest of training. For monkeys, the initial bias may result from a center preference influenced by the behavioral set up, where monkeys were seated in a primate chair that was centered to the middle of the display panel. As the task became more difficult, monkeys became increasingly engaged with the visual stimulus, were able to override their initial spatial predispositions, and bias disappeared. The opposite happened for the rats: As the task became more difficult, they disengaged from the visual stimulus and their spatial predispositions largely determined their behavior. Indeed rats have a well-documented thigmotaxis, i.e., preference for walls, or what Small referred to as a “…thygmotactic rat-hole psychosis” (Small, 1901; p. 229), manifested here by their strong center avoidance. Not unlike rats, tree shrews also developed a spatial bias in the more difficult contrast conditions, although smaller in magnitude and of opposite sign, i.e., center preference as also seen in monkeys. Since bias limits behavioral performance, this may explain why tree shrews showed lower maximum performance than did the monkeys. Notably, tree shrews showed no spatial bias in the initial training sessions, a behavior that has been previously described for this species (Leonard et al., 1966; Fobes and King, 1978). One might conjecture that rats have been unfairly penalized by the 3AFC task with left, center, and right response locations, since task performance is vulnerable to the inherent avoidance of open spaces characteristic of rat behavior. It is certainly possible that for a different spatial configuration, i.e., a circular testing chamber or vertically aligned response locations, rat performance could have been improved. Our conclusions on performance thus apply strictly to the specific task that we have employed, such that our results can serve as a baseline for future studies on comparative visual learning. We cannot rule out that visual task designs may exist, for which rats perform equally well, or better than the other species tested here, but this would need to be demonstrated in future experiments.

### Behavioral strategies

Repeating responses at a previously rewarded response location is a common source of behavioral error in laboratory tasks. When the reward is randomized, as in most behavioral tasks including ours, basing decisions on previous reward history is an ineffective strategy. Similarity in the proportion of win stay versus lose stay responses is indicative of an animal that is not relying on reward toward the solution of the task. This is what is seen in both the tree shrews and the monkeys, they are equally likely to repeat responses at a given location whether or not that location was previously rewarded. Here rat behavior departed from that of the other species, as their behavioral responses were, to a large degree, determined by reward history. Rats almost never returned to the center position following an unrewarded response at that position. However, prior reward at the center position allowed the rat to overcome its center avoidance, at least in the easier contrast conditions. In this regime, the rats are using their reward history to compensate for their inherent spatial bias. As the task became more difficult, rats abandoned this reward dependent compensation, and their behavior became determined almost entirely by their innate spatial bias.

As Thorndike noted in his law of original behavior, “… to any situation an animal will, apart from learning, respond by virtue of the inherited nature of its reception-, connection- and action-systems.” (Thorndike, 1911; p. 243). The central assumption in behavioral neuroscience has long been that extinction of these initial schemata is critical to discrimination learning and that once learning has occurred a largely analogous circuitry is engaged across a broad spectrum of vertebrate species. Here, we provide evidence that contradicts this assumption. The inherent schemata do not simply vanish but are rather incorporated into the behavioral strategy used toward optimizing reward in the task. This has important implications because species-specific predispositions and bias continue to manifest themselves following learning, animals must therefore engage species specific circuits during task performance. Our results highlight the necessity of careful comparative studies in translational neuroscience.

## Synthesis

Reviewing Editor: Tatiana Pasternak, University of Rochester Decisions are customarily a result of the Reviewing Editor and the peer reviewers coming together and discussing their recommendations until a consensus is reached. When revisions are invited, a fact-based synthesis statement explaining their decision and outlining what is needed to prepare a revision will be listed below. The following reviewer(s) agreed to reveal their identity: Rufin Vogels, Andreas Nieder. Note: If this manuscript was transferred from JNeurosci and a decision was made to accept the manuscript without peer review, a brief statement to this effect will instead be what is listed below.

Both reviewers found the paper interesting and felt that it provides an valuable contribution to our understanding of species differences in problem solving. However, there was an agreement among the reviewers that it remains unclear whether the inferior performance in rodents reflects the peculiarities of the 3AFC discrimination task, since it may not match their response predisposition or whether it represents a failure of rats to learn this visual discrimination.

This issue was initially raised by Reviewer 1. Reviewer 2 agreed that the revision needs to include the discussion of this issue.

In your revision please discuss this issue and the potential limitations of the task. Also, address the few minor comments raised by Reviewer 1.

-----------

REVIEWER 1

Advances the Field (Required)

This is a difficult one. This study shows that rats learn a particular contrast/flicker discrimination task much slower than primates. They also show that rats develop a strong response bias in that particular task when it gets difficult, reflecting a disposition to avoid a centrally positioned response keys. This is useful information for rodent researchers who are interested to use this discrimination task. However, it is unclear whether these results reflect the peculiarities of the implemented task or a failure in general to learn this sensory discrimination in general. As long as this is not solved, this experiment does not learn us a lot except that rats perform/learn poorly in the sort of 3AFC task employed in this study.

Comments to the authors

The authors show that rats learn a 3AFC contrast/flicker discrimination much slower than primates. I argued before that this difference in performance does not learn us much except that the 3AFC task the authors employed is not optimal for rodents. The authors reply by saying that 3AFC is not "unusual in visual psychophysics". However, the issue is not about the 3AFC task, it is about how it was implemented with a spatial response. The authors show that the rats suffer from a strong spatial response bias, a tendency to avoid the central response key. If they had implemented their task with a single central lever (go - nogo) or response targets above each other, the results might have been different. I am still convinced it is essential to adapt the task to the behavioral response predispositions of the species and using the exactly the same task in difference species does not make much sense if one is interested in sensory processing. This limits the use of these data to make an inference about the use of rodents for translational studies. The authors should discuss this issue and limitation of their study.

Except for the main point above, I am satisfied with their revisions. There remain a couple of minor points that should be addressed by the authors.

1. The authors should provide the number of trials that each of the 3 species performed (lines 89 and following). Also, they should put the p values between brackets.

2. Line 149: which "rule" are the authors referring to?

3. I like Table 1 since it provides individual animal data. These data on interindividual differences should be described in the text.

REVIEWER 2

Advances the field

I think this is an interesting and timely paper. It shows profound performance differences in the three animal species from different branches of the phylogenetic tree. The paper therefore is a welcome addition to the field of comparative psychology and neuroscience. Beyond that, and as the authors emphasize, such differences in problem solving across species need to be taken into account also in translational research.
